# MRD in Venetoclax-Based Treatment for AML: Does it Really Matter?

**DOI:** 10.3389/fonc.2022.890871

**Published:** 2022-07-18

**Authors:** Massimo Bernardi, Felicetto Ferrara, Matteo Giovanni Carrabba, Sara Mastaglio, Francesca Lorentino, Luca Vago, Fabio Ciceri

**Affiliations:** ^1^ Istituto di Ricovero e Cura a Carattere Scientifico (IRCCS) San Raffaele Scientific Institute, Department of Onco-Hematology, Milan, Italy; ^2^ Antonio Cardarelli Hospital, Division of Hematology, Naples, Italy; ^3^ Vita-Salute San Raffaele University, Milano, Italy

**Keywords:** acute myeloid leukemia, venetoclax, MRD - measurable residual disease, treatment, low-intensity

## Abstract

The prognosis of newly diagnosed patients with acute myeloid leukemia is still unfavorable in the majority of cases within the intermediate and mainly adverse genetic risk group but also in a considerable fraction of favorable-risk patients, mainly due to recurrence of disease after complete remission achievement or, less frequently, primary refractoriness. Besides genetic classification at diagnosis, post-treatment prognostic factors include measurable residual disease evaluation in patients in complete remission and in most cases measurable residual disease (MRD) positivity predicts hematologic relapse potentially allowing early therapeutic intervention. Currently, the most commonly used methods for detection of minimal residual disease are multiparameter flow cytometry and quantitative PCR, applicable to around 90% and 50% of patients, respectively. In addition, in > 90% of acute myeloid leukemia (AML) patients, molecular aberrations can be identified by next-generation sequencing, a technology that is widely used in clinical practice for the initial mutational screening at the time of diagnosis but more often, for MRD detection because its flexibility allows almost every mutated gene to be used as an MRD marker. Threshold levels of residual disease and correlation with outcome have been thoroughly studied and established in younger patients treated with intensive induction and consolidation chemotherapy as well as after allogeneic transplantation. Yet, experience on MRD monitoring and interpretation in patients treated with low-intensity regimens, including new agents, is still limited. The updated armamentarium of anti-leukemic agents includes the BCL-2 inhibitor venetoclax, which demonstrated good tolerability, high response rates, and prolonged overall survival when combined with hypomethylating agents or low dose cytarabine in patients considered elderly/”unfit” to tolerate intensive regimens. Although remissions with negative minimal residual disease clearly translated into improved outcomes after intensive treatments, data supporting the same evidence in patients receiving low-intensity venetoclax-based treatments are not still consolidated. We here review and discuss more recent data on the minimal residual disease interpretation and role in AML patients treated with venetoclax-based combinations.

## Introduction

**Figure 1:**
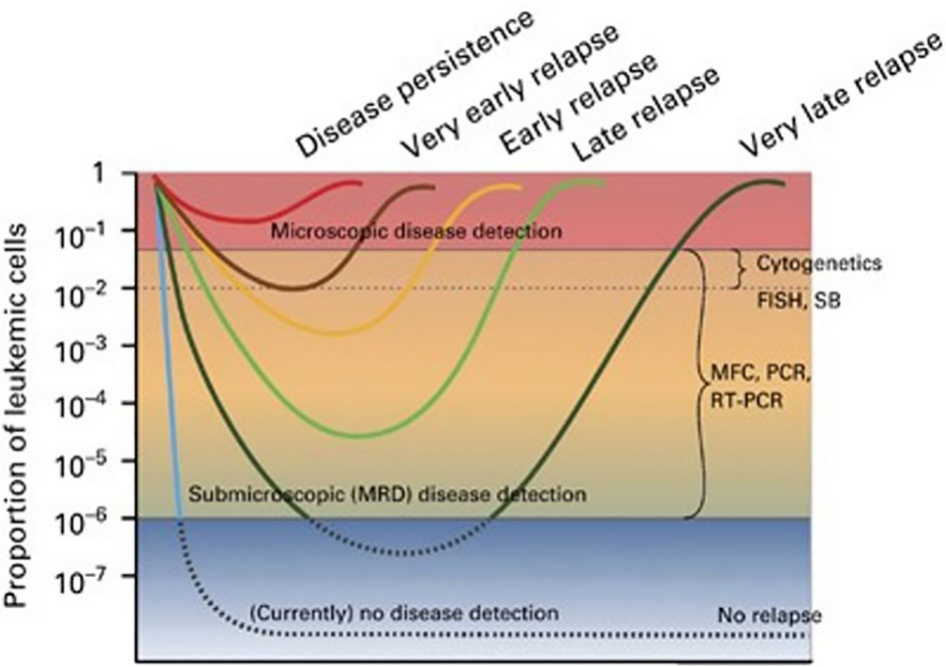
The concept of MRD. Hypothetical scenarios of leukemia cell burden changes in response to therapy (8). Figure from Buckley SA, et al. BMT 2013 (8).

**Figure 2:**
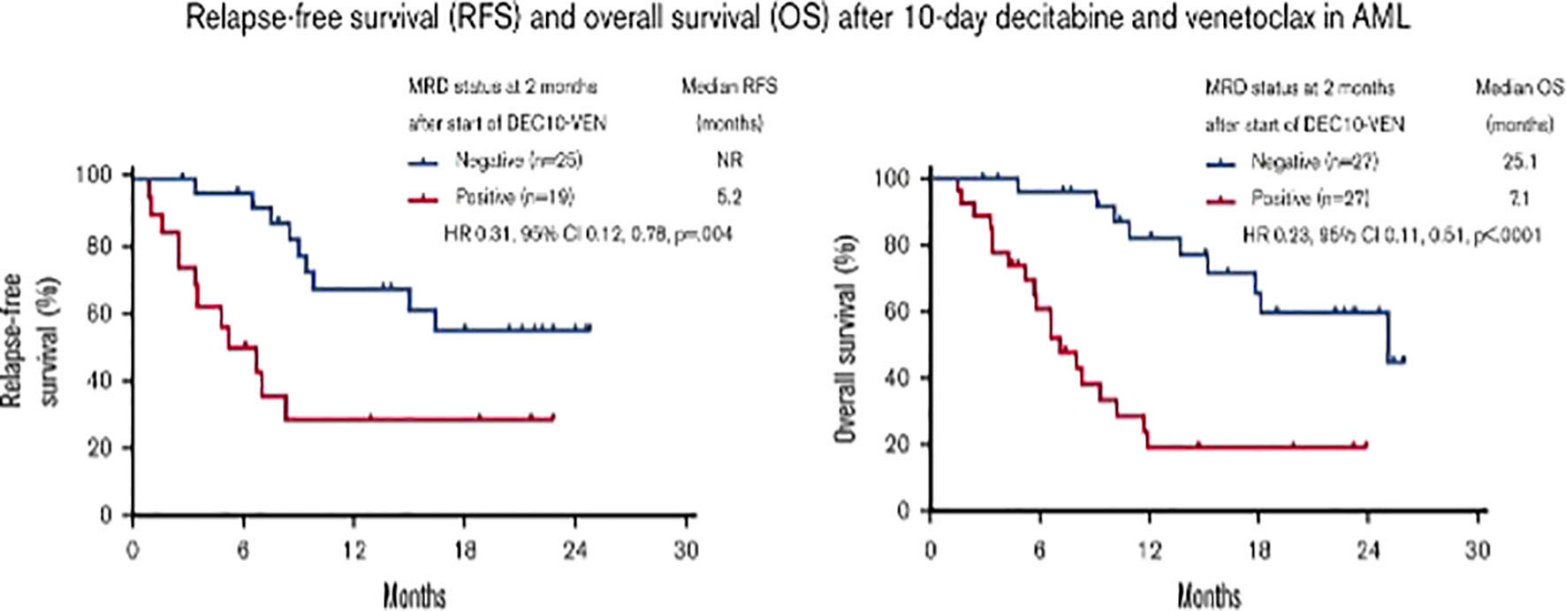
Prognostic value of measurable residual disease after venetoclax and decitabine in acute myeloid leukemia (10). Figure from Maiti A, et al. Blood Adv 2021 (10). HR, hazard ratio; NR, not reached.

Acute myeloid leukemia (AML) is an extremely heterogeneous disease with variable characteristics and prognosis, driven by several biological factors of the malignant cells comprising different combinations of genetic mutations and chromosomal aberrations, as well as abnormal expression of cell surface antigens (1, 2). The detection of one or more such abnormalities, which are present in more than 90% of cases, has prognostic implications at diagnosis, during treatment, and follow-up of patients in morphological complete remission (CR). Identification of low levels of leukemic cells in the bone marrow (BM) or peripheral blood (PB), below the sensitivity of conventional microscopic examination, has been termed minimal or better measurable residual disease (MRD) and showed to correlate with an increased risk of relapse. In fact, whereas AML remission after initial treatment can be obtained in most patients, relapse invariably occurs if any trace of resistant disease is not eradicated after therapy (3–7) (**Figure 1**). Currently, the most common methods to study MRD are multiparameter flow cytometry (MFC) and reverse transcriptase quantitative polymerase chain reaction (rqPCR). New molecular (MOL) methods, such as next generation sequencing (NGS) and digital PCR (dPCR), are still under validation for this purpose and are not widely available **Table 1**). MRD assessment is recognized as an important predictor for risk stratification and therapeutic decision-making in newly diagnosed AML patients receiving intensive therapy programs, whereas evidence supporting the use of MRD as a predictive biomarker in patients treated with low-intensity approaches, including new agents, is still limited. This probably reflects the fact that most “unfit” patients were not expected to obtain deep remissions with low-intensity available treatments, such as the hypomethylating agents (HMA) azacytidine (AZA) and decitabine (DEC), and with low-dose cytarabine (LDAC), at least until the advent of venetoclax (VEN). VEN is a highly potent, specific BCL-2 inhibitor with limited monotherapy activity in AML. It has been investigated in prospective trials in combination with AZA, DEC, or LDAC demonstrating tolerability, higher response rates, and longer overall survival (OS) than single-agent treatments in elderlyunfit” patients. Outcome data of these studies have also suggested that MRD-negative CR (CRMRD-) could be predictive of improved OS (9, 10) (**Figure 2**). Following initial favorable results with VEN-HMA and VEN-LDAC, new low-intensity combinations, including VEN, have been proposed in prospective studies which also evaluate the MRD response. Among them, a phase II study at the MD Anderson Cancer Center recently has showed that the cladribine/LDAC combination plus VEN, alternating with AZA plus VEN, is a low-intensity regimen which is effective and well-tolerated among older patients (≥60 years), producing high response rates with durable CRMRD- by MFC-MRD (11). Overall, data on utility of MOL-MRD as a prognostic factor in these patients are still limited. VEN has been also studied in combination with intensive induction and consolidation chemotherapy in younger patients with untreated and relapsed or refractory (R/R) AML. High rates of CR and CRMRD- have been obtained, with most patients being able to receive an allogeneic stem cell transplantation (alloSCT) (12). While recommendations for MRD evaluation in these patients are the same as after any standard intensive treatment of AML, the main question currently is: does MRD monitoring matter to predict the outcome of AML patients receiving lower-intensity VEN-based therapies? Moreover, is MRD a reliable surrogate end point for studies exploring the benefit of these treatments? In this review we summarize the application and interpretation of MRD analysis to predict and monitor the outcome of AML patients under VEN-based treatments.

**Table 1:** Comparison of different approaches to analyze MRD (46).

	MFC	rqPCR/ddPCR	NGS
**Detects**	Immunophenotypically abnormal cell populations	Single molecular abnormality	Multiple molecular abnormalities.
**Advantages**	- Applicable to >90% of cases; - Identifies abnormal stem/progenitor cell compartment; - Easily quantified; - Sensitive; - Quick; - Can assess hemodilution; - Distinguishes between live and dead cells; - Can identify targets for immunotherapy.	- Reproducible; - Highly sensitive; - Can identify therapeutic targets; - Easily quantified and standardized.	- Applicable to >90% of cases; - Can identify therapeutic targets; - Platform can be standardized.
**Disadvantages**	- Not all AMLs have abnormal immune phenotype; - Phenotype may change over time; - Sensitivity is not uniform between patients; - Best results require fresh material; - Experienced personnel required; - Analysis/data interpretation have subjective elements; - Difficult to standardize.	- Not widely applicable; - Genetic abnormalities can persist, even in long-term remission; - Genetic clonal heterogeneity; - Genetic evolution over time; - Emergence or selection of sub-clone(s) at relapse.	- Requires error correction to overcome low sensitivity; - Mutated genes are also present in healthy individuals; - Genetic clonal heterogeneity; - Genetic evolution over time; - Emergence or selection of sub-clone(s) at relapse; - Bioinformatic approaches are not uniform.

Table reproduced from Walter R., oral presentation (46).

## Prognostic Impact of MRD in AML

MRD evaluation has acquired the strong ability to predict therapeutic decisions for, and the outcomes of AML patients. The recently updated recommendations of the ELN AML-MRD working group provide guidance in harmonization, refinement, and validation of MRD testing in patients who achieve morphologic remission with full or partial hematologic recovery (Composite CR [CRc]: complete remission [CR] + CR with incomplete hematologic recovery [CRi]) (13). MRD studies have had a major impact on expert panel guidelines, in particular those directed to the management of favorable and intermediate ELN risk patients in CRc with persistent MRD positivity at the end of standard consolidation. These patients are at a high risk of relapse and should be offered an alloSCT, followed whenever possible, by maintenance treatment. Differently, patients with the same ELN prognostic risk in CR and with undetectable MRD have a high probability of being cured after chemotherapy programs (14, 15) (**Figure 1**). Two meta-analyses, which reported a relationship between achievement of MRD negativity and superior survival in patients with AML, provided important prognostic information for patients receiving an alloSCT, guiding post-transplant therapeutic decisions. The results suggest a strong relationship between pre-alloSCT MRD status and post-alloSCT relapse and survival. Conversely, the possible benefits of CRMRD+ conversion to CRMRD- before transplant have to be demonstrated by prospective studies as available data suggest that the post-transplant Graft-versus-Leukemia (GVL) effect is similar in MRD-positive and MRD-negative patients (16–18). Overall, MRD evaluation is prognostically relevant in AML patients receiving intensive treatment programs. However, 25%-30% of MRD-negative patients relapse. Thus, MRD is not yet predictive for the individual patient and its use has to be improved and validated to better personalize post-remission treatments.

## MRD Studies in AML Patients Receiving VEN-Based Treatments

As previously introduced, there is little data on the clinical significance of MRD in AML patients receiving low-intensity prolonged treatments. Among low-intensity approaches, VEN-based combinations with a HMA have significantly improved the outcomes of patients “unfit” for intensive chemotherapy and alloSCT. The results of the phase 3 VIALE-A trial have demonstrated that the combination of VEN and AZA significantly improves either the rate of CRc and MFC-MRD remission as compared to AZA alone, in elderlyunfit” patients with untreated AML. Pratz et al. have recently confirmed the importance of MFC-MRD, after a longer follow-up of patients included in the same study (median > 20 months): those who achieved CRc and CRMRD- by MFC also showed a longer duration of remission (DoR), OS, and event-free survival (EFS) than patients in CRc and with MFC-MRD positivity (19, 20). In the VEN containing arm, 164 out of 190 (86%) patients with CRc were evaluable for MRD by MFC. CRMRD- was achieved by 67 out of 164 (41%), and 97 out of 164 (59%) had CRMRD+. Median DoR, EFS, and OS were not reached in patients with CRMRD- and were 9.7, 10.6, and 18.7 months, respectively, in CRMRD+ patients. MOL-MRD was evaluated by NGS in 100 patients: CRMRD- was obtained in 50% of patients with FLT3 mutations, 49% of patients with IDH1/2 mutations, 30% of patients with TP53 mutations, and 88% of patients with NPM1mut. Multivariate analysis showed that CRMRD- was a strong predictor of OS (HR 0.285). Of interest, 25% of patients achieved CRMRD- by the end of cycle 1, 27% between cycle 2 and cycle 4, with a further 21% achieving a response thereafter. Thus, the timing of MRD response may be independent of the time of achievement of hematological remission and may occur well after the patient has achieved the CR. Taken together, data from VIALE-A study suggested that MRD response, at any time, during treatment could predict OS in a context of low-intensity and prolonged treatment (20). Another analysis of the prognostic value of achieving CRMRD- was conducted in 83 “unfit” AML patients obtaining a CRc after first-line therapy with 10-day DEC plus VEN. Relapse free survival was longer in 52 CRMRD- patients compared with 31 CRMRD+ (not reached vs 5.2 months) (10). Molecular patterns of response and treatment failure after frontline VEN combinations in older patients with AML have been studied: primary and adaptive resistance have been most commonly characterized by acquisition or enrichment of clones activating signaling pathways such as FLT3 or RAS or biallelically perturbing TP53 (21). This raised the issue of designing new strategies/combinations to target clonal evolution. Of note, the study presented at the 63rd° (2021) meeting of the American Society of Hematology, combining cladribine/LDAC/VEN alternating with AZA/VEN, produced astounding results in elderly AML patients: CRc rate was 93% (CR 80%) and treatment related mortality was 2% (1 patient). Of 51 evaluable patients in CRc after 1-2 cycles, 43 (84%) were negative for MFC-MRD (11). Strategies, including the adjunct of a third agent to VEN and HMA, are under investigation to evaluate the possible benefit on MRD negativization and patients’ outcome. Noteworthy among published studies, Di Nardo et al. combined VEN and the FLAG-Ida regimen attaining the CRMRD- in 96% of untreated AML and 69% of R/R AML, and 1-year OS for transplanted patients of 94% and 78%, untreated and R/R, respectively (12). Similarly, another single-center, single-arm, phase 2 trial evaluated VEN plus intensive chemotherapy with CLAD, idarubicin, and cytarabine in patients with untreated AML or high risk MDS. This combination induced a high rate of molecular remission: 37/45 patients (82%). In this study, after a median follow-up of 13.5 months, the median DoR, EFS, and OS were not reached. Moreover, durable MRD-negative remissions were obtained across all the prognostic subgroups (22).

## Timing for MRD Testing in Patients Receiving Low-Intensity, VEN-Based Treatments

**Figure 3:**
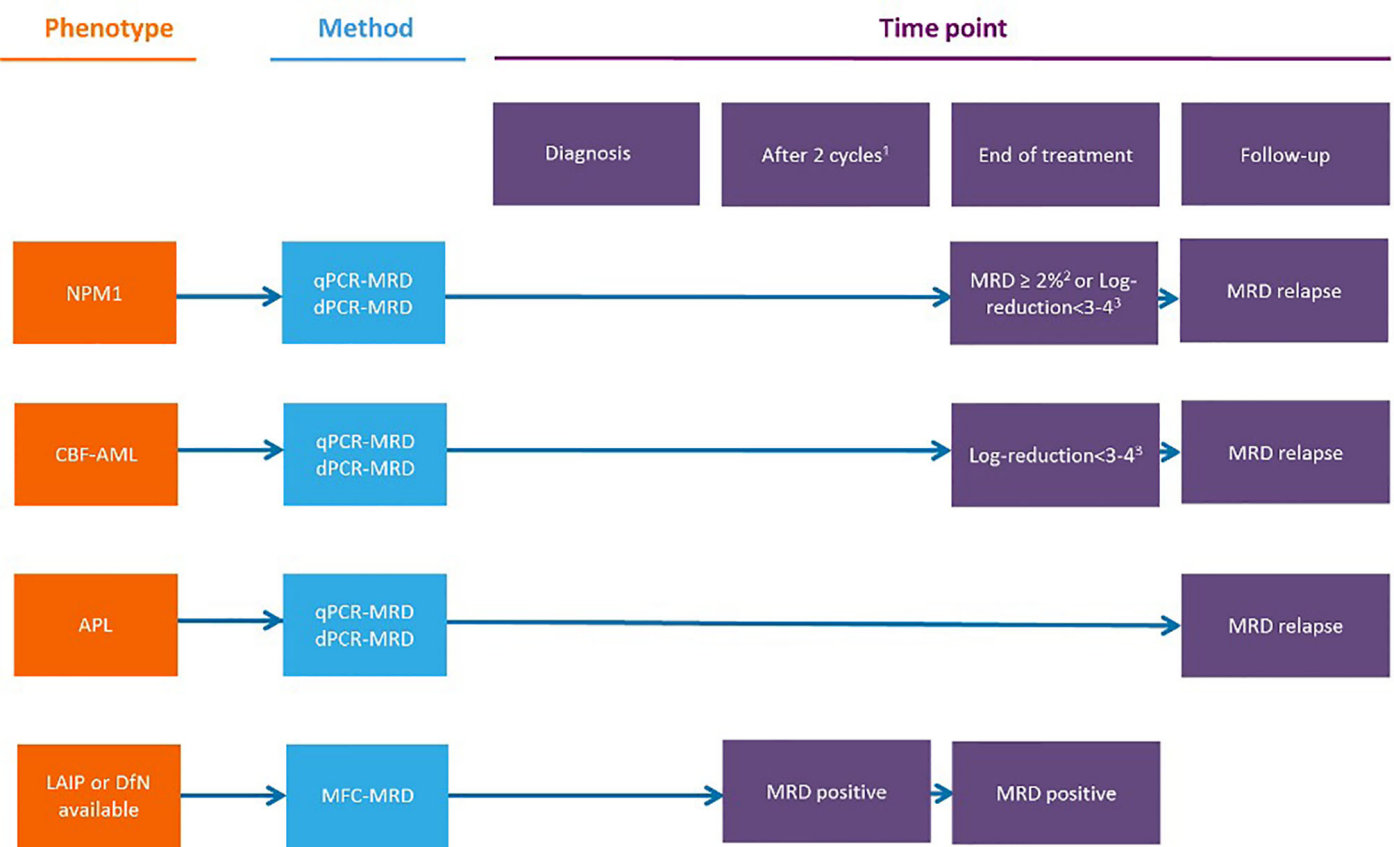
Measurable residual disease response and prognosis in treatment-naıve acute myeloid leukemia with venetoclax and azacytidine. **(A)** DoR among patients with composite complete remission. **(B)** Forest plot for DoR in subgroups (20). Figure from Pratz KW, et al. JCO 2021 (20). CR, complete remissions; CRi, complete remission with incomplete hematologic recovery; DoR duration of remission; MRD, measurable residual disease; NR, not reached.

The appropriate timing for MRD monitoring during and after therapy for AML patients largely depends on several variables, among which are the subset of the disease, the chance to follow MRD with a MOL-based and/or MFC-based technique, the type of therapy (e.g., intensive vs. non intensive, targeted), and the consolidation with alloSCT. The recently updated ELN recommendations published in 2018 precisely suggest how MRD should be assessed in AML patients receiving intensive treatments (3, 13) (**Figure 3**). Moreover, many studies highlight the prognostic value of MFC-MRD and MOL-MRD analysis before and after alloSCT (23–30). Less is known regarding the optimal timing for MRD detection during lower-intensity therapies that usually envisage continuative cycles, until failure, and potentially induce different kinetics of disease response, as compared to conventional chemotherapy. Specifically, the most relevant clinical trials using VEN adopted different timeline strategies for MRD monitoring. In the VIALE-A trial, BM assessments were performed at initial screening at the end of first VEN-AZA cycle, and every three cycles thereafter, until two consecutive samples confirmed a CRc; patients with CRc and evaluable for MFC-MRD had a median of 3.0 (range: 1.0-8.0) MRD assessments. Notably, of 67 out of 164 patients reaching the CRMRD-, only 25% obtained MRD negativity after cycle 1 and 27% by the end of cycle 4, whereas 49% of patients became MRD-negative after further cycles. For patients who attained the CRMRD- at any time, the median and the 12-month DoR, EFS, and OS were not reached at 81.2%, 83.2%, and 94.0%, respectively. Patients who achieved the CRMRD- after cycle 1, or thereafter, had a similar significantly better 12-month OS than CRMRD+ patients. Importantly, among authors’ observations, the MRD response may occur independently of, and well after achievement of the clinical remission (20). Maiti et al. at the MDACC (MD Anderson Cancer Center) studied VEN combined with 10 days of DEC in older/”unfit” patients with untreated AML, followed by VEN plus 5-day DEC every 4-6 weeks. Eighty-three out of 97 patients achieved CR/CRi, and 52 (54%) became MRD-negative by MFC, within 2 months (median, range 0.9-3.1 months). BM samples were evaluated at the end of cycles 1, 2, and 4. The authors found that outcomes according to MRD status at 2nd and 4th time points were similar, irrespective of transplantation. Moreover, patients who obtained the CRMRD- within 4 months had longer OS, EFS, and relapse free survival (RFS), and attainment of CRMRD- after cycles at 1 and 2 was associated with significantly better OS in patients with intermediate- and adverse-risk cytogenetics (10). Other reports are available on this field which include small numbers of patients with no pre-defined time points for MRD follow-up. A French center retrospectively studied 19 consecutive untreated AML elderly/”unfit” patients, who received VEN-AZA or VEN-LDAC, monitored by MFC-MRD after each of first 3 cycles, then in different way for each patient. MRD negativity was obtained in 9 out of 11 tested patients in CRc (81.8%). Patients with persistent CRMRD- had a longer DoR than those with detectable MRD at even only one assessment at any time point during follow-up (31). A Chinese group that tested VEN in association with low dose DEC as post alloSCT maintenance for 20 high-risk AML and MDS patients, chose to perform a BM evaluation, with assessment of MRD monthly for the first six months of therapy and every two months or with longer intervals thereafter. Also, only one CRMRD+ patient and one CRMRD- patient relapsed after alloSCT (32).

## MRD as a Surrogate End Point to Establish Response to Low-Intensity, VEN-Based Treatments

The ultimate goal of all oncology drugs is to improve patient-centered “hard” endpoints, such as OS, quality of life, or both. However, clinical trials may take up to 10 years to demonstrate a benefit for an experimental drug when the primary endpoint is survival and may request a huge number of patients “needed-to-treat”, with considerable effects on cost requirements. For such reasons, there is a need for alternative (surrogate) endpoints, which can give the same information on treatment effect earlier than the primary endpoint. A surrogate end point has been defined as an alternative endpoint (such as a biological marker, physical sign, or precursor event) that can be used as a substitute for a clinically meaningful endpoint that measures how a patient feels or survives (33). Using surrogate endpoints to measure whether a new drug works can facilitate faster access to new therapies. In recent years, in the cancer therapeutic area, surrogate endpoints accounted for almost 80% of all clinical studies supporting regulatory approvals (34), partly as the result of an increased use of “expedited” regulatory pathways for the approval of drugs by both United States and European medical agencies (35, 36). To be useful, a surrogate endpoint should be strongly associated with the true outcome, lie in the causal pathway for the definitive outcome, should manifest early in the course of follow-up, and should be relatively easy to measure (37). Several methods have been developed to assess the predictive value of a surrogate endpoint. However, the method most suited for regulatory approval is trial-level surrogate validation. Trial-level validation occurs by plotting a change in the surrogate against the change in the hard endpoint across several randomized studies. Each trial serves as one data point. A linear regression analysis is then performed to see if a correlation exists between a change in the surrogate and in the hard endpoint, the coefficient of determination of this linear regression provides a measure of strength of the association between the effects. This measure is termed R-trial and suggests validating a surrogate endpoint if the value is sufficiently close to one (38–41). It is notable that for most approvals, no R-trial can be calculated because no validation study has ever been done for MRD as surrogate endpoint. In AML patients MRD would be an ideal candidate to be a surrogate endpoint of survival benefit, although difficulties lie in verifying how the direction and relative magnitude of treatment effect on MRD is reproduced on the definitive outcome of OS. MRD may fail in its ability to predict hard endpoints for technical factors in measuring this surrogate that introduces uncertainty and irreproducibility among clinical trials and could weaken a direct causal link between the surrogate and the hard endpoint. Arguments in favor of using MRD status as a surrogate endpoint include a recent meta-analysis of 81 publications and 11,151 patients with untreated AML, treated with induction and consolidation chemotherapy, which suggested that achievement of MRD negativity has prognostic importance in AML and may be a valid surrogate marker for both DFS and OS, irrespective of age, AML subtype, sample type, time of MRD assessment, and MRD detection method (17). Additionally, the long-term follow-up of AML patients in CR after chemotherapy enrolled in the QUAZAR AML-001 trial of maintenance with oral AZA, showed an association between post-induction MRD positivity with significantly shorter OS and RFS (42). The role of MRD testing in older or “unfit” patients treated with lower intensity regimens is less explored. The study published in 2021 by Maiti A. et al. is the first report on the association of MRD response and survival benefit in AML patients treated with VEN-based low-intensity regimens (10) (**Figure 2**). Although available data suggest that there is a place for MRD as a surrogate endpoint to guide treatment choices in AML, further validation with prospective investigation is warranted, in particular, in patients receiving lower-intensity treatments.

## Discussion

What is the value of detecting a deep remission in AML? Although most AML patients achieve a CR after induction chemotherapy, post-remission treatments to prevent relapse are required. Selecting the optimal consolidation, particularly for patients with intermediate risk AML, remains a challenge. Reports from trials and real life suggest that MRD, measured by any methodology (MFC/MOL), is an important biomarker for improving prognostics, monitoring, and efficacy-response assessments during morphological remission (3). In particular, MRD analysis has become crucial in identifying, among patients obtaining a CR, a subgroup with poor prognosis after initial chemotherapy. Despite the huge work of the ELN MRD panel, further standardization of MRD assays and analytical tools are still needed to permit the potential for a greater predictive power (13). Variation in methods and lack of standardization limits the comparability of MRD assessments between different laboratories. For instance, reproducibility of NGS, across laboratories, has not been evaluated although it remains a promising method for MRD analysis. Regarding the combination of MOL and MFC assays, some studies have shown that each technique has an independent and additive prognostic value for predicting the rate of relapse and survival in younger treatment-naıve AML patients treated with intensive regimens. Further studies are needed to integrate the results of multiple MRD assays into one prognostic score (13, 43). The U.S. Food and Drug Administration (FDA) has recognized MRD as a potential surrogate end point for outcomes, although this was from mostly non-randomized trial data, and thus provided guidance that can be used in prospective trials and to accelerate new drug development (5, 44). Importantly, no randomized comparison has yet been performed between conventional treatments or alloSCT in MRD positive intermediate-risk group patients and MRD validity in the post-transplant setting has not been validated. For this reason, ELN guidelines recommend that all AML clinical trials include MRD monitoring at any BM evaluation during treatment and follow-up of patients in CR (13). Also, the prognostic relevance of MRD in non-intensive AML treatments has not been established. The important results obtained with low-intensity, VEN-based combination therapies, in elderly/”unfit” AML patients, have raised the question if MRD-negative remissions could also translate into improved outcomes in this setting. The VIALE-A trial has provided evidence that MRD negativity can be obtained in an estimated one-fourth of patients who obtained a CRc with the VEN-AZA combination. A recent *post-hoc* analysis from this trial has confirmed the potential of MRD as an important disease response measure (19, 20). Since MRD analysis was not continuously and sequentially performed, the most informative time point(s) for MRD assessment could not definitely be established. Still, late MRD-negative responses have been obtained and have been associated with a better outcome. Therefore, first MRD evaluations may not be indicative of the full effect of the VEN-AZA treatment. Then, as MRD-negative responses occur over time during a continuum of care, quantitative MRD determinations should provide greater information relevant to the risk of relapse when performed over multiple time points to establish any possible change in tumor burden. Taken together, all available data support the conclusion that in the lower intensity, the VEN-based treatment setting of monitoring the MRD response could be useful in predicting the survival and the risk of relapse of patients achieving CRc. Otherwise, low-intensity treatments including VEN, offered to older/”unfit” patients, have offered an improvement in survival and quality of life compared to standard care, which although impressive in some cases, does not translate to evidence of a long-term cure.

Then, why should MRD studies be pursued in a population of “unfit” patients with no curative perspectives? The majority of these patients, indeed, are expected to continue the ongoing low-intensity treatment until its failure and/or unacceptable toxicity. In the daily practice, some patients manifest perplexity and doubts because of the possibility of never stopping the treatment. Therefore, a major issue related to this and other similar approaches in clinical hematology is the possibility of treatment discontinuation, without affecting the prognosis. Achieving MRD negativity could become a goal for CRMRD+ patients, as the results from ongoing investigations that address the issue of treatment deintensification or discontinuation in CRMRD- patients identify a subset of cases with long-term EFS in this context (20). Recent observations suggest that the risk of relapse and duration of RFS and OS were similar between two small cohorts of patients in which, due to different reasons, some of them stopped the therapy. Factors favoring sustained treatment free remission within the “stopped” cohort included NPM1 and/or IDH2 mutation at diagnosis, MRD-negative CR, and at least 12 months of VEN-based combination therapy prior to discontinuation (45). Confirmation of these findings in a larger patient population is strictly required before adopting such an approach in daily practice and a prospective randomized discontinuation study would definitely clarify this important issue. Conversely, some patients initially considered “unfit” for intensive treatment (mainly because of the disease itself) can become “fit” on disease remission achievement, since performance status, instrumental activities of daily living, infections, and organ functions significantly improved. These patients can be readdressed to consolidation with alloSCT if indicated, according to the prognostic risk of the disease. Reaching the CRMRD- is supposed to be a favorable prognostic factor for patients undergoing alloSCT after initial treatment with VEN-HMA combinations, as demonstrated in patients receiving intensive treatments before transplant (10). For this reason, achievement of CRMRD- has been included as one of the objectives of clinical trials of patients with high-risk AML, studying the VEN combination with intensive chemotherapy followed by alloSCT. Finally, future analysis should define which end point(s) could actually be recognized to qualify MRD as a surrogate endpoint in clinical trials exploring low-intensity treatments. For instance, the decrease of MRD levels during treatment, achievement of MRD negativity, and frequency of MRD-negativity in patients.

## Conclusions

Achievement of CRMRD- in AML patients treated with VEN-based combinations is associated with improved survival. However, the use of MRD as a surrogate endpoint in these patients requires further validation, possibly with randomized studies, to establish its definitive role in clinical management and relapse prediction. Long-term MRD monitoring during treatment or follow-up should be based on individual clinical features. Studies of therapy deintensification/discontinuation in the MRD-negative subset could further enlarge the body of evidence of the clinical benefit of MRD monitoring.

## Author Contributions

MB wrote the first draft of the manuscript, the abstract, the introduction and discussion sessions. FF, MC, SM, FL, and LV wrote sections of the manuscript. FC and FF reviewed the paper. All authors contributed to manuscript revision, read, and approved the submitted version.

## Conflict of Interest

The authors declare that the research was conducted in the absence of any commercial or financial relationships that could be construed as a potential conflict of interest.

## Publisher’s Note

All claims expressed in this article are solely those of the authors and do not necessarily represent those of their affiliated organizations, or those of the publisher, the editors and the reviewers. Any product that may be evaluated in this article, or claim that may be made by its manufacturer, is not guaranteed or endorsed by the publisher.
